# Admixture with indigenous people helps local adaptation: admixture-enabled selection in Polynesians

**DOI:** 10.1186/s12862-021-01900-y

**Published:** 2021-09-22

**Authors:** Mariko Isshiki, Izumi Naka, Ryosuke Kimura, Nao Nishida, Takuro Furusawa, Kazumi Natsuhara, Taro Yamauchi, Minato Nakazawa, Takafumi Ishida, Tsukasa Inaoka, Yasuhiro Matsumura, Ryutaro Ohtsuka, Jun Ohashi

**Affiliations:** 1 Department of Biological Sciences, Graduate School of Science, The University of Tokyo, Tokyo, 113-0033 Japan; 2 Department of Human Biology and Anatomy, Graduate School of Medicine, University of the Ryukyus, Nishihara, 903-0125 Japan; 3 Genome Medical Science Project, Research Center for Hepatitis and Immunology, National Center for Global Health and Medicine, Chiba, 272-8516 Japan; 4 Graduate School of Asian and African Area Studies, Kyoto University, Kyoto, 606-8501 Japan; 5 Department of International Health and Nursing, Faculty of Nursing, Toho University, Tokyo, 143-0015 Japan; 6 Faculty of Health Sciences, Hokkaido University, Sapporo, 060-0812 Japan; 7 Graduate School of Health Sciences, Kobe University, Kobe, 654-0142 Japan; 8 Department of Human Ecology, Faculty of Agriculture, Saga University, Saga, 840-8502 Japan; 9 Faculty of Health and Nutrition, Bunkyo University, Chigasaki, 253-8550 Japan; 10 Japan Wildlife Research Center, Tokyo, 130-8606 Japan

**Keywords:** Positive selection, Admixture-enabled selection, Admixture, Rapid adaptation, Population genomics, Genetic ancestry, Polynesia

## Abstract

**Background:**

*Homo sapiens* have experienced admixture many times in the last few thousand years. To examine how admixture affects local adaptation, we investigated genomes of modern Polynesians, who are shaped through admixture between Austronesian-speaking people from Southeast Asia (Asian-related ancestors) and indigenous people in Near Oceania (Papuan-related ancestors).

**Methods:**

In this study local ancestry was estimated across the genome in Polynesians (23 Tongan subjects) to find the candidate regions of admixture-enabled selection contributed by Papuan-related ancestors.

**Results:**

The mean proportion of Papuan-related ancestry across the Polynesian genome was estimated as 24.6% (SD = 8.63%), and two genomic regions, the extended major histocompatibility complex (xMHC) region on chromosome 6 and the ATP-binding cassette transporter sub-family C member 11 (*ABCC11*) gene on chromosome 16, showed proportions of Papuan-related ancestry more than 5 SD greater than the mean (> 67.8%). The coalescent simulation under the assumption of selective neutrality suggested that such signals of Papuan-related ancestry enrichment were caused by positive selection after admixture (false discovery rate = 0.045). The *ABCC11* harbors a nonsynonymous SNP, rs17822931, which affects apocrine secretory cell function. The approximate Bayesian computation indicated that, in Polynesian ancestors, a strong positive selection (*s* = 0.0217) acted on the ancestral allele of rs17822931 derived from Papuan-related ancestors.

**Conclusions:**

Our results suggest that admixture with Papuan-related ancestors contributed to the rapid local adaptation of Polynesian ancestors. Considering frequent admixture events in human evolution history, the acceleration of local adaptation through admixture should be a common event in humans.

**Supplementary Information:**

The online version contains supplementary material available at 10.1186/s12862-021-01900-y.

## Background

The human occupation of Oceania began approximately 47,000 years ago [38]. The first immigrants settled Sahul, a continent that comprised the land masses of present-day Australia, New Guinea, and the surrounding small islands. They are considered the ancestors of modern Papuans and Aboriginal Australians. They colonized the islands of New Britain and New Ireland, reaching the Solomon Islands by 28,000 years ago [65]. This region of initial colonization is known as Near Oceania. Probably due to the large expanse of ocean to the east of Near Oceania, Remote Oceania, which includes eastern part of the Solomon Islands, Vanuatu, Fiji and all the islands of Polynesia, remained unoccupied until the Late Holocene.

Austronesian (AN)-speaking people from Southeast Asia who possessed the advanced navigation skills necessary for a long-distance voyage first colonized Remote Oceania. They are called the Lapita people after their culture, Lapita, which is characterized by pottery decorated with distinctive motifs. Remains of their characteristic pottery suggest that they originated in Taiwan and arrived in the Bismarck Archipelago about 3500 years ago [6, 28, 57]. Lapita people then expanded into Remote Oceania using their advanced navigation skills. They reached western Polynesia, Tonga and Samoa, 2900‒2700 years ago [9, 52, 53], and finally Hawaii, Easter Island and New Zealand by 1200–800 years ago [4, 17, 19]. They are considered the direct ancestors of modern Polynesians.

Several genetic studies have found that about 20–30% of the modern Polynesian genome was derived from Papuan-related ancestry, and the rest was derived from Asian-related ancestry [24, 27, 56, 66], indicating that the Asian-related Polynesian ancestors admixed with indigenous people in Near Oceania, the Papuan-related ancestors, during their expansion from Near Oceania to Polynesia. The admixture between Asian- and Papuan-related ancestors has been estimated to have occurred about 3000 years ago [47, 66].

*Homo sapiens* populations have experienced admixture many times during the species’ expansion and adaptation all over the world [16]. Admixture with populations from different genetic backgrounds would lead to introduction of adaptive genetic variants at intermediate frequencies in the gene pool, and thus can enable rapid adaptation to the local environments. Such admixture-mediated adaptation must have played an important role in human evolution. In the case of Polynesians, their Papuan-related ancestors, who had inhabited Oceania for tens of thousands of years, might already have had some genetic components adapted to Oceanian environment at the time of admixture. Therefore, it is expected that if the Polynesian ancestors had acquired genomic regions adaptive to the Oceanian environment through admixture, the frequency of those regions would increase by natural selection, and the regions would contribute to the adaptation of Polynesians. The phenomenon whereby genome regions introduced through admixture increase the fitness of admixed populations is called “adaptive introgression”. This phenomenon is well-studied in the case of the admixture between modern humans and archaic hominins, such as Neanderthals and Denisovans [50], and adaptive introgression between modern human populations, often called as admixture-enabled selection, has also been studied in a few specific groups [7, 8, 12, 15, 21, 22, 37, 42, 44, 54, 60, 68]. Recently African ancestry enrichment around the human leukocyte antigen (*HLA*) region and signals of polygenic selection on immune function were observed in Latin American populations, suggesting admixture drove rapid adaptive evolution in human populations [37]. Investigating the effect of admixture on local adaptation in Polynesian genomes is therefore important for deepening our understanding of human evolution.

Previously we detected the signatures of local selective sweeps in Polynesian genomes by comparing the haplotype variation between Tongans and reference populations [27]. In this study, local ancestry was estimated across the genome in the same Polynesian subjects to reveal the effect of admixture on their local adaptation. Genomic regions with particularly high levels of Papuan-related ancestry in Polynesian genomes could have undergone admixture-enabled selection, based on the principle that the proportions of local ancestry are expected to be similar across the genome, unless affected by natural selection. We detected the signatures of admixture-enabled selection from Papuan-related ancestors in two genomic regions (chromosomes 6 and 16) in Polynesians. One of the regions harbored the *ATP-binding cassette transporter sub-family C member 11* (*ABCC11*), and the *ABCC11* allele (rs17822931-C) which determines wet earwax, was likely to have experienced a strong positive selection in Polynesians after the admixture. Our results suggest that the admixture with Papuan-related ancestors contributed to the rapid adaptation of Polynesian ancestors to the environment of Oceania.

## Results

### Clustering analysis and Admixture proportion

**Fig. 1:**
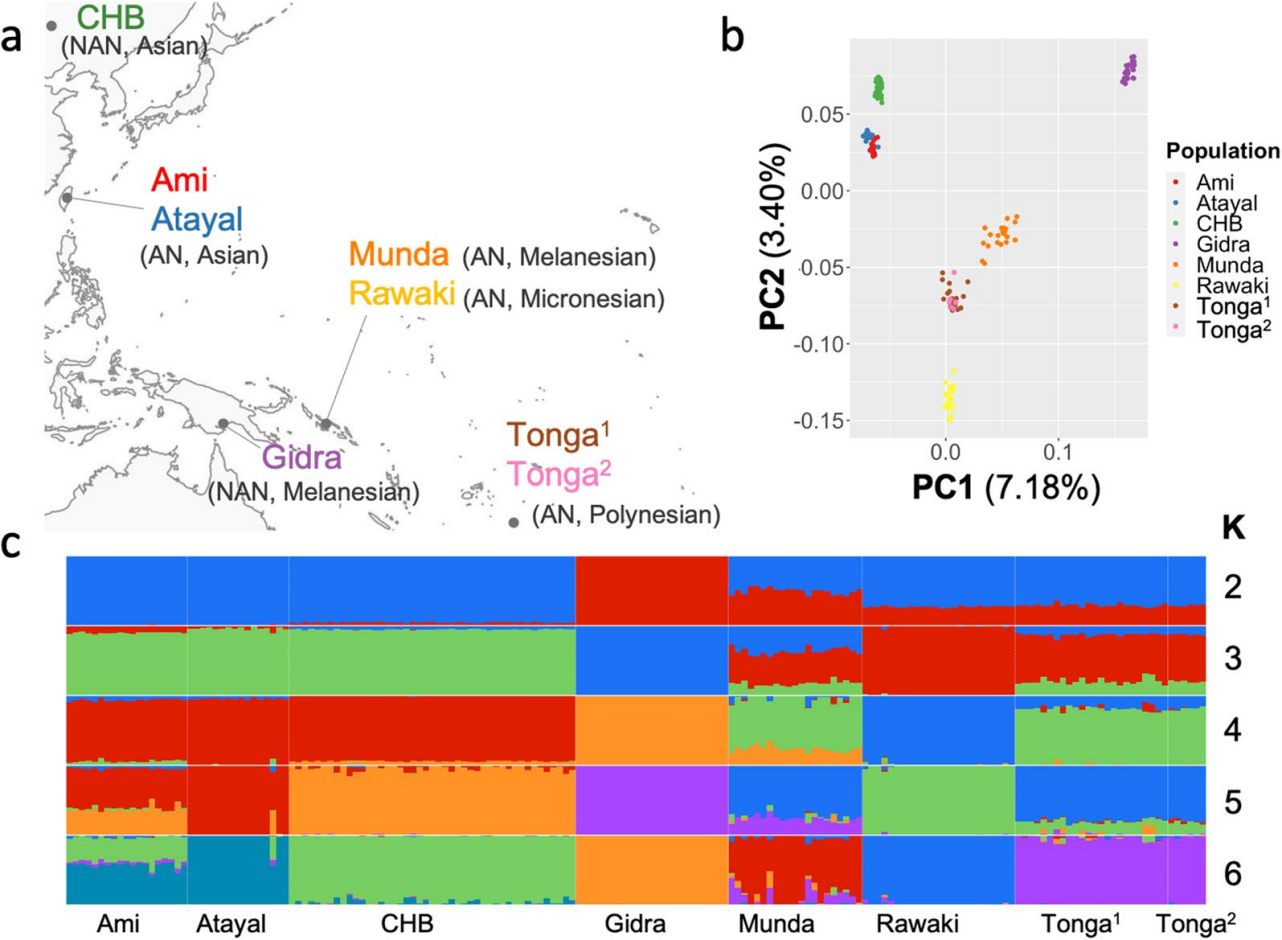
PCA and ADMIXTURE analysis of eight populations. **a** A map of eight populations analyzed in this study. AN: AN-speaking population. NAN: NAN-speaking population. **b** PCA plot for the eight populations. **c** Results of ADMIXTURE analysis for K ranging from 2 to 6. The lowest cross-validation error was obtained for K = 5. The map depicted in Fig. 1 (**a**) was taken from FREEWORLDMAP.NET (https://www.freeworldmaps.net/). 1 Tongans obtained from Kimura et al. [27]. 2Tongans obtained from Qin and Stoneking [48] and Pugach et al. [46]

Principal component analysis (PCA) and ADMIXTURE analysis [2] were performed on 179 individuals from five Oceanian and three Asian populations (Fig. 1). Percentages of variance was 7.18% for PC1 and 3.40% for PC2 (Fig. 1b). AN-speaking admixed populations (i.e. Munda, Rawaki and Tongans) were plotted between Papuans (i.e. Gidra) and Asians (i.e. CHB, Ami and Atayal) as expected from their population histories. Two Tongan populations obtained from different studies were clustered. Figure 1c illustrates individual ancestry proportion inferred by ADMIXTURE analysis for numbers of postulated ancestral populations (K) ranging from two to six. K = 5 provided the lowest cross-validation error (Additional file 1: Fig. S1). Assuming red and blue components for K = 2 as Asian- and Papuan-related ancestries, respectively, the proportion of Asian-related ancestry for Tongans was estimated as 71.4% (SD = 2.21%).

The f3 statistics for Tongans were estimated using the Gidra as a proxy for Papuan-related ancestors and the Han Chinese population of Beijing (CHB) [61] or Aboriginal Taiwanese [30, 46, 48] for Asian-related ancestors, to examine whether they descended from a mixture of the two ancestral populations. Concordant with previous studies, negative f3 statistics were observed regardless of which populations were assumed as a proxy for Asian-related ancestors, indicating that Tongans or Polynesians were the descendants of a mixture of Papuan- and Asian-related ancestors (Table 1).

**Table 1:** Results of 3-Population test for Tongans

Papuan-related ancestors	Asian-related ancestors	f3	SE	Z	SNPs
Gidra	Taiwan	−0.0174	0.000737	−23.7	49523
Gidra	CHB	−0.0108	0.000753	−14.3	49523

SE, standard error

The proportion of Asian-related ancestry in Polynesian genomes was estimated using the f4 ratio test, assuming the phylogeny shown in Additional file 1: Figure S2. The proportion of Asian-related ancestry was estimated as 67.4% (SE = 1.22%, Z = 55.2).

### Natural selection acted on the genomic regions derived from Papuan-related ancestors in Polynesians

**Fig. 2:**
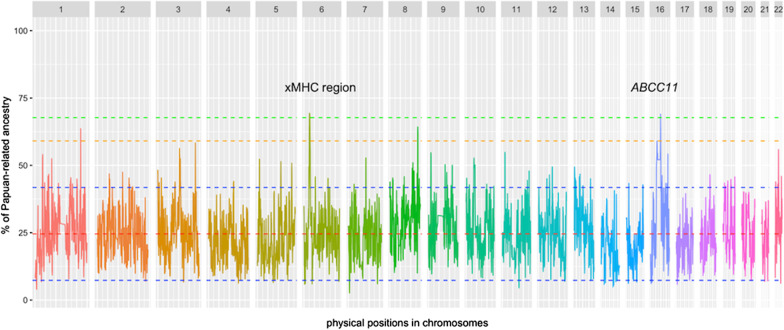
Proportion of Papuan-related ancestry across Polynesian genome. Each color represents a different chromosome. Red dashed line represents the genome-wide mean. Blue, orange, and green dashed lines represent 2 SD, 4 SD, and 5 SD from the mean, respectively. Proportions of Papuan-related ancestry were deviated from the mean by more than 5 SD in genomic regions on chromosomes 6 (xMHC region) and 16 (*ABCC11* gene)

The contributions of Asian- and Papuan-related ancestry across the Polynesian genome (Papuan versus Asian ancestry) were measured using the Effective Local Ancestry Inference (ELAI) algorithm [15] with CHB and Gidra as proxies for Asian- and Papuan-related ancestors, respectively. Figure 2 shows the mean proportion of Papuan-related ancestry across the Polynesian genome, estimated by the ELAI program with 100 admixture generations. The mean proportion of Papuan-related ancestry was estimated as 24.6% (SD = 8.63%), which corresponded with the previous studies [24, 66]. Two genomic regions displayed proportions of Papuan-related ancestry more than 5 SD greater than the mean (Fig. 2) (chr6:26471596–28011652 and chr16:48226479–48266831). The detected regions on chromosome 6 and 16 contained 51 SNPs and 4 SNPs, respectively. The high Papuan-related ancestry region on chromosome 6 (Papuan-related ancestry proportion: 67.72–69.37%) was located within the extended major histocompatibility complex (xMHC) region. 43 genes such as histone protein genes and DNA-binding protein genes are clustered within the region. Since this region contained 51 SNPs in strong LD, the candidate genes cannot be detected from the current dataset. The high Papuan-related ancestry region on chromosome 16 (Papuan-related ancestry proportion: 67.85–69.03%) contained only the *ABCC11* gene.

Since local ancestry inference results can be very sensitive to the choice of source populations, we performed ELAI analysis assuming Aboriginal Taiwanese, who are considered to be closer to Asian-related ancestors of Polynesians than CHB are, as a proxy for Asian-related ancestors. The two genomic regions were also detected in the ELAI analysis (Additional file 1: Fig. S3), suggesting that the high Papuan-related ancestry regions did not come from the differentiation between CHB and Asian-related ancestors of Polynesians.

A recent study demonstrated that Polynesian genomes contained European and Native American ancestries [20]. Native American ancestry was observed in Eastern Polynesians while European ancestry was observed in all Polynesian populations analyzed in the study. Thus, we conducted ELAI analysis with three-way admixture model assuming CEU from1000 Genomes Project Phase 3 [1] as a proxy for European-related ancestors. Although a relatively small contribution from European-related ancestors was observed, the highest degrees of Papuan-related ancestry were also detected in the two genomic regions detected above (Additional file 1: Fig. S4).

### Coalescent simulations

To examine whether genetic drift alone could cause genomic regions to display proportions of Papuan-related ancestry more than 5 SD greater than the mean, ELAI analysis was conducted for the whole-genome data generated by coalescent-based simulations, assuming selective neutrality (see Methods for details). As shown in Additional file 1: Fig. S5, the mean and SD of Papuan-related ancestry estimated from the simulation data were similar to those of real data. To evaluate the false discovery rate (FDR) of our approach, we performed 100 independent runs of coalescent-based simulation and subsequent ELAI analysis, and then counted the number of independent genomic regions showing Papuan-related ancestry more than 5 SD above the mean for each simulation run. Out of the 100 simulation runs, no genetic regions showed a proportion of Papuan-related ancestry more than 5 SD above the mean in 91 runs, and the excess was detected in a single genomic region in each of the remaining 9 runs. The threshold (mean + 5sd) used in this study therefore seemed to yield a family-wise error rate (FWER) of 0.09. Under the condition of FWER = 0.09, our results corresponded to FDR of 0.045 (= 0.09/2), since two regions showed such deviations in the real genotype data. Thus, the xMHC and *ABCC11* regions could be shaped by genetic drift, but are more likely to have been shaped by positive selection since the admixture of the Papuan- and Asian-related ancestors of modern Polynesians.

### Selection acted on the earwax-associated SNP (rs17822931) on *ABCC11*

**Fig. 3:**
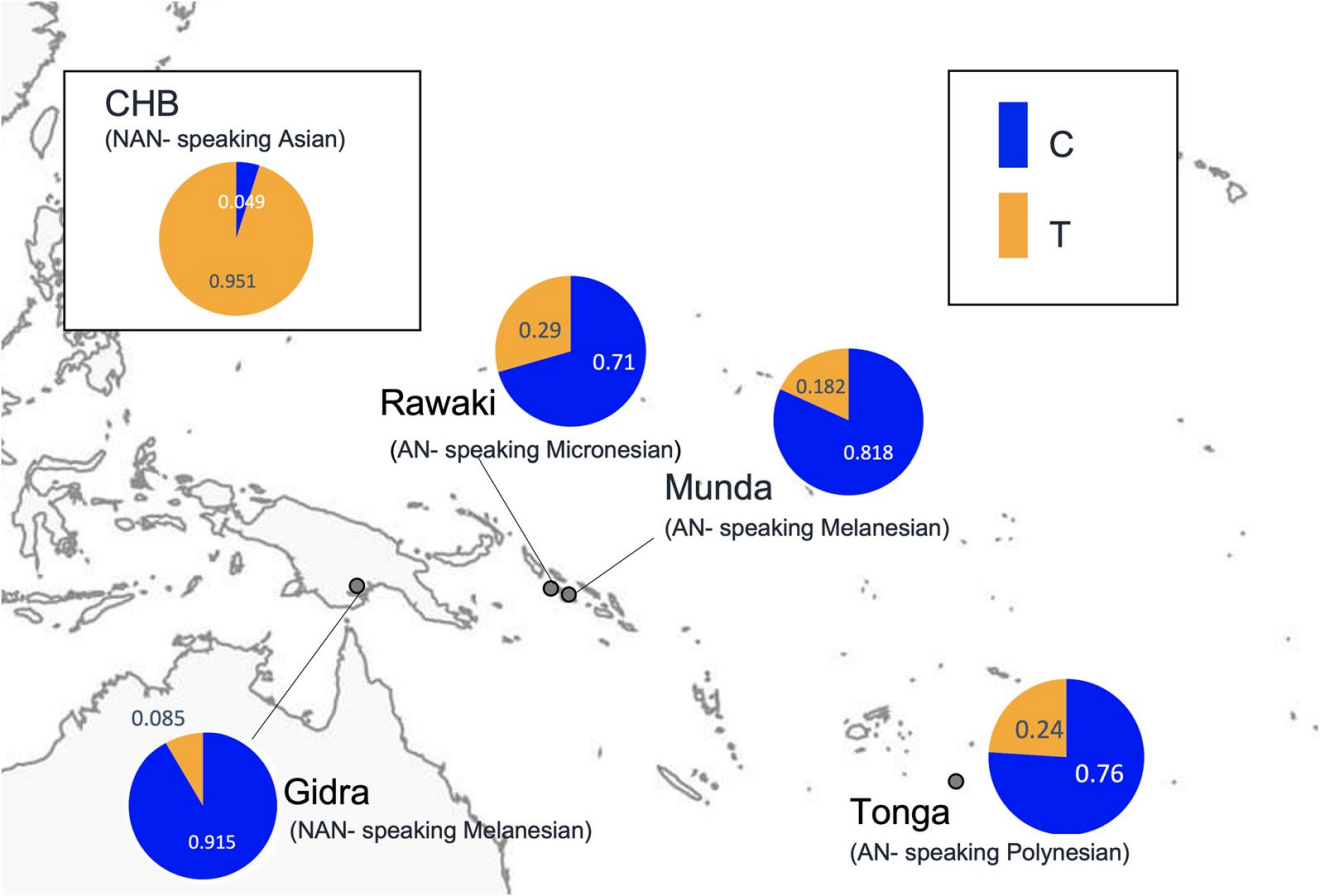
Distribution of rs17822931 in Oceanian populations. Allele frequencies of rs17822931-C (*ABCC11* allele for wet-type earwax) and -T (*ABCC11* allele for dry-type earwax) for four Oceanian populations genotyped in this study. The allele frequency in 40 individuals from HapMap CHB was added for comparison. The map depicted in Figs. 3 was taken from FREEWORLDMAP.NET (https://www.freeworldmaps.net/)

To evaluate the intensity of positive selection on the *ABCC11* gene, we focused on a nonsynonymous SNP (G180R), rs17822931. This SNP is known to affect apocrine secretory cell function and determine phenotypes such as earwax type and body odor [34, 67]. The derived allele of rs17822931, rs17822931-T or 180R, associated with a dry type of earwax and a reduction in body odor, was frequently observed in Northeast and East Asia [34, 67]. The allele frequencies in four Oceanian populations and 40 individuals of HapMap CHB are shown in Fig. 3 and Table 2. The ancestral allele, rs17822931-C or 180G, was found to be dominant in Oceanian populations.

**Table 2:** Frequency and LD statistics of rs12445647-T and rs17822931-C

Populations	Region	n	Frequency		D'	R^2^
			rs12445647-T	rs17822931-C		
Tonga (Polynesian)	Oceania	174	0.60	0.76	0.98	0.44
Munda (Melanesian)	Oceania	170	0.68	0.82	1	0.47
Rawaki (Micronesian)	Oceania	107	0.62	0.71	1	0.67
Gidra (Papuan)	Oceania	165	0.80	0.92	0.95	0.34
YRI^a^	Africa	216	0.07	1	NA	NA
CEU^a^	Europe	198	0.08	0.87	1	0.01
CHB^b^	Asia	40	–	0.05	–	–
CHB^a^	Asia	206	0.01	0.03	1	0.16
JPT^a^	Asia	208	0	0.12	NA	NA
CHS^a^	Asia	210	0.01	0.16	1	0.05
CDX^a^	Asia	186	0.08	0.46	1	0.1
KHV^a^	Asia	198	0.09	0.36	1	0.16

^a^Individuals from 1000 Genomes project Phase 3

^b^40 individuals from HapMap project used in ELAI analysis

The approximate Bayesian computation with a forward-time simulation was conducted to estimate the selection coefficient, *s*, for rs17822931-C in Tongans. Here, only simulation runs that resembled the observed allele frequency in Tonga when the run was terminated were accepted. The distribution of *s* in 10,000 accepted runs is shown in Additional file 1: Fig. S6. The mean of *s* was 0.0217, and the 95% credible interval was 0.0124–0.0309.

### Origin of rs17822931-C in Polynesians

Next, to confirm if the rs17822931-C allele in Tongans or Polynesians originated from Papuan-related ancestors, rs12445647 was genotyped as a tag SNP for rs17822931-C derived from Papuan-related ancestors. As shown in Table 2, rs12445647-T was observed at a high frequency in Oceanian populations compared to the other populations. More importantly, the *r*^2^ value, a measure of linkage disequilibrium (LD), between rs12445647-T and rs17822931-C was higher in Polynesian and Papuan populations than those in Asian populations. Such higher LD shared with modern Polynesian and modern Papuan populations implies that haplotype harboring rs12445647-T and rs17822931-C in Polynesians originated mainly from Papuan-related ancestors.

### Haplotype and expression level of *ABCC11*

The rapid increase in allele frequency of rs17822931-C in Polynesian ancestors might be due to the fact that the haplotype harboring rs12445647-T and rs17822931-C, which is thought to have been introgressed from Papuan-related ancestors to the Polynesian ancestors, is functionally different from the haplotype harboring rs12445647-G and rs17822931-C. Since the amino acid in position 180 of the ABCC11 protein is glycine in both haplotypes, there seems to be no difference in the protein function. We therefore examined the possibility that the *ABCC11* mRNA expression level was different between the haplotype harboring rs12445647-T and rs17822931-C and the haplotype harboring rs12445647-G and rs17822931-C, using publicly available data: genotype data of the 1000 Genomes Project Phase 3 populations [1] and microarray data of the HapMap3 populations [13, 18, 59] obtained from the ArrayExpress database at EMBL-EBI. Since no significant association of rs12445647 with the *ABCC11* expression level was observed in 217 unrelated subjects with the rs17822931-CC genotype (Additional file 1: Fig. S7), haplotype harboring rs12445647-T and rs17822931-C, originated from Papuan-related ancestors, does not seem to be a special haplotype that affects the expression level of the *ABCC11* gene.

## Discussion

The results of PCA and ADMIXTURE were consistent with previous studies, suggesting that most fraction of Polynesian genomes were derived from Asian-related ancestry [24, 27, 46, 56, 66]. The f3 statistics revealed that Polynesians experienced admixture between Papuan- and Asian-related ancestors (Table 1). The proportion of Asian-related ancestry was estimated as 71.4% and 67.4% by ADMIXTURE (K = 2) and the f4 ratio test, respectively. The mean proportion of Asian-related ancestry across the Polynesian genome was estimated to be 75.4% (SD = 8.63%) in ELAI analysis. The estimates were equivalent to the proportions estimated by STRUCTURE analysis [45] in our previous study [27]. Based on these results, the proportion of Asian-related ancestry in the Polynesian genome is expected to fall within 70–80%.

Two genomic regions, xMHC and *ABCC11*, with proportions of Papuan-related ancestry greater than 5 SD above the mean were detected in this study (Fig. 2). To examine the possibility that the excess of Papuan-related ancestry was an artifact of the reference populations assumed, we used other reference populations to confirm the results. The genomic regions of xMHC and *ABCC11* were detected even when Aboriginal Taiwanese were used as a proxy for Asian-related ancestry and when the recent contact of Europeans was assumed (Additional file 1: Figs. S3 and S4). Thus, it is plausible that the detected regions were not an artifact of the reference populations assumed. However, we cannot deny the possibility that the excess of Papuan-related ancestry came from the differentiation between the Lapita and the current East Asian populations as the Lapita people, the direct Asian-related ancestors of Polynesians, were distinct from extant East Asian populations today [56]. A coalescent simulation assuming selective neutrality suggested that these regions were likely to have been subjected to admixture-enabled selection (i.e., The admixture with Papuan-related ancestors have contributed to the rapid local adaptation of Polynesian ancestors). One of the two candidate regions of admixture-enabled selection was located in the xMHC region. This region overlapped the candidate regions for selective sweeps identified in our previous study with the same genotype data of Tongans [27]. Genes involved with various biological processes such as the immune response and epigenetic regulation of gene expression clustered within the region. However, a single candidate polymorphism subjected to positive selection was difficult to be identified from many SNPs that exist in the region due to strong LD between them. Thus, in this study, we focused on the other region, where only the *ABCC11* gene is located as a protein-coding gene. This region was not detected in our previous scan for positive selection using LD-based methods [27]. A number of LD-based methods, such as REHH [55], iHS [63], rMHH and rHH [26], have been developed to detect signatures of recent positive selection acting on beneficial derived allele. In the LD-based methods, the ratio of the degree of LD (e.g., extended haplotype homozygosity in REHH) between the derived allele and the ancestral allele is expressed as a test statistic. The test statistic for beneficial derived allele is expected to be larger than that for neutral derived allele at the same frequency, since the former exhibits higher degree of LD. LD-based methods are suited for detecting recent positive selection acting on derived allele, but would not show high statistical power for that on ancestral allele in an admixed population, since haplotypes harboring the ancestral allele have already had many recombination events before the admixture in ancestral populations and no extended LD is observed even if the ancestral allele rapidly increases its frequency after the admixture. This is thought to be the main reason that the positive selection that acted on the *ABCC11* gene was not detected in our previous study [27].

A nonsynonymous SNP of *ABCC11*, rs17822931, which affects apocrine secretory cell function and determines the type of earwax and body odor an individual produces, exhibited a large difference in frequency between East Asians and Oceanian populations (Fig. 3). Analysis of the tag SNP, rs12445647, suggested that the majority of rs17822931-C alleles in Polynesians originated mainly from Papuan-related ancestors, not from Asian-related ancestors (Table 2). Therefore, the frequency of rs17822931-C had been low in Polynesian ancestors at the time of admixture and has been rapidly increased due to positive selection since then. The rs17822931-T, a derived allele of rs17822931, has been shown to have experienced positive selection in East Asians as an adaptation to a colder climate, with a selection coefficient estimated as approximately 0.01 [26, 39]. The selection coefficient of rs17822931-C was estimated to be 0.0217 in Polynesians (Additional file 1: Fig. S6), indicating that the positive selection acting on the ancestral allele in Polynesian ancestors was stronger than that on the derived allele in East Asians. To the best of our knowledge, this study is the first report of positive selection having acted on rs17822931-C, an ancestral allele associated with the production of wet earwax.

The frequency of rs17822931-T shows the north–south gradient in East Asia [67]. Considering that rs17822931-T experienced positive selection in northeast Asia [39], it is likely that the observed frequency gradient along the latitude may be a result of the shifting balance of which allele was selected more strongly in each environment (e.g. climatic conditions, temperature and pathogen prevalence). However, the evolutionary significance of rs17822931 is still unknown. One possible explanation for positive selection acting on rs17822931-C in Polynesian ancestors is the association of rs17822931 with the amount of apocrine colostrum secretion [36]. Colostrum has an important role in the development of the immune system in newborns [62]. Women with the rs17822931-C allele are significantly less likely to lack colostrum and can produce significantly more colostrum than women without rs17822931-C [36]. Since various pathogens were present in tropical region, colostrum may have been important until the development of modern medical technologies. Genes involved in immune function often present strong signatures of selection and admixture-enabled selection against immune-related genes has been reported in admixed populations [7, 8, 12, 15, 21, 22, 42, 44, 49, 54, 60, 68]. Consistently, as xMHC region also contains genes involved in immune responses against pathogens, infectious disease is a possible driver of the selection in these regions. However, as well as the high apocrine secretion, the ABCC11 wild-type protein has a function to transport various substrates such as bile acids, conjugated steroids, and cyclic nucleotides [11]. Since these substrates were involved with various physiological processes, further investigation is necessary to clarify the driving force of the positive selection acted on the *ABCC11* in Polynesian ancestors.

Two recent ancient DNA studies suggested that the first immigrants into Remote Oceania almost entirely lacked Papuan-related ancestry components [31, 56]. Thus, it is possible that admixture occurred after the settlement of Polynesian ancestors in Remote Oceania dissimilar to the postulated population history in this study that Polynesian ancestors got admixed with Papuan-related ancestors before the expansion into Remote Oceania. Even though the location and timing of the admixture event may need further investigation, there is no doubt that admixture-enabled selection has occurred in Polynesian ancestors after the admixture with Papuan-related ancestors.

## Conclusions

In this study, we detected two genomic regions subjected to admixture-enabled selection in Polynesians. It is considered that de novo mutations adaptive to the environment generally takes much longer time to reach high frequencies. Therefore, for Asian-related ancestors of Polynesians, it would have been advantageous to acquire pre-existing genetic materials through admixture with Papuan-related ancestors who had already adapted to over tens of thousands of years. The acceleration of adaptation is also observed in Latin American populations [37]. As *Homo sapiens* have experienced admixture many times in the last few thousand years [16], admixture-enabled selection should be a common event in humans.

## Methods

### Subjects and data

A genome-wide SNP dataset comprising 24 Tongan individuals, AN-speaking Polynesians living in Ha’apai Island and Nuku'alofa of the Kingdom of Tonga, and 24 individuals from Gidra, NAN-speaking Melanesians (Papuans) in the lowlands of Western Province, Papua, New Guinea [27], 21 individuals from Munda, AN-speaking Melanesians in the New Georgia Islands in the western part of the Solomon Islands [21], 24 individuals from Rawaki, AN-speaking Micronesians migrated from the overpopulated Gilbert Islands (Kiribati) to the New Georgia Islands in the 1960s. All individuals were genotyped using Affymetrix GeneChip® Human Mapping 250 K Nsp SNP array. After merged with the HapMap genotype data of 45 unrelated individuals from CHB [61], the dataset consisted of 231,049 autosomal SNPs.

Since Lapita people, the direct ancestors of Polynesian people, are suspected to have originated in Taiwan, the other dataset which includes Aboriginal Taiwanese was also prepared. 35 Aboriginal Taiwanese (16 individuals from Atayal and 19 individuals from Ami) and six Tongans [30, 46, 48] were added to the above dataset. After SNPs with a genotyping rate lower than 0.95 were filtered out, 179 individuals and 49,523 SNPs were left.

### PCA and ADMIXTURE

A principal component analysis (PCA) and ADMIXTURE analysis were performed on the second dataset comprising of 179 individuals and 49,523 autosomal SNPs. PCA was conducted with using PLINK software v1.90b5.2 [10]. ADMIXTURE analysis was carried out using ADMIXTURE version 1.3.0 [2] for different values of K (from K = 2 through K = 6). Cross-validation procedure implemented in ADMIXTURE package was performed to find the best value of K. The results were drawn using pophelper R package 2.3.0 [14].

### 3-population test and F4 ratio test

The 3-population test and f4 ratio test were conducted on the dataset which contained Aboriginal Taiwanese using the AdmixTools package version 4.1 [43]. As an outgroup for the f4 ratio test, the HapMap data of 60 unrelated individuals from Yoruba in Ibadan, Nigeria (YRI) [61] were also merged. After merging all the datasets, those SNPs with a genotyping rate lower than 0.95 were filtered out, leaving 49,523 SNPs to be used.

The f3(C; A, B) statistics should be negative if a population C has descended from a mixture of populations A and B. Assuming CHB or Taiwanese as a proxy of Asian-related and Gidra as that of Papuan-related ancestors, f3(Tonga; CHB or Taiwanese, Gidra) was calculated to test if admixture occurred in the ancestors of Tonga population.

An f4 ratio test, assuming the population relationships shown in Additional file 1: Fig. S2, was performed to estimate the proportion of admixture. The proportion of Asian-related and Papuan-related ancestry, α and 1-α, respectively, was estimated by computing the ratio of two f4 statistics:

α = f4(CHB, YRI; Tonga, Gidra)/f4(CHB, YRI; Taiwan, Gidra) [43, 51].

### Genome-wide scan for natural selection

To detect signals of natural selection having acted over the genomic regions derived from Papuan-related ancestors in Polynesian genomes, ELAI analysis [15] was preformed across the genome for 23 Tongan subjects assuming CHB and Gidra as a proxy for their Asian- and Papuan- related ancestors, respectively. The ELAI method computes expected ancestry dosage at each marker for each individual using a two-layer hidden Markov Model. To exclude close relatives, the Identical-By-Descent (IBD) value of each pair of individuals was checked. The calculation of IBD values was performed after LD pruning using PLINK software v1.90b5.2 [10] with the following settings, which define window size, step and the *r*^2^ threshold: –indep-pairwise 50 5 0.5. Since one pair of individuals showed an IBD value higher than 0.125 (IBD value = 0.5185), one individual from this pair was excluded from the following analyses.

A total of 162,358 autosomal SNPs that showed a genotyping rate higher than 0.95 and that were polymorphic in each population were used for ELAI analysis. ELAI analysis was performed with the ELAI version 1.00 software with settings which defined the number of EM steps as 20, the upper layer number of clusters as 2, and lower layer number of clusters as 10, in accordance with the manual [15]. Since the ancestors of Polynesians were considered to have reached Oceania about 3000 years ago, the admixture generations were set as 100, which is consistent with the dates of admixture for Polynesian populations estimated in the previous studies: 83 (95% CI 66–112) generations [46], 90 generations (95% CI 77–131) [47], and 99 generations (95% CI 19–267) [66]. Statistical analysis was conducted using R version 3.5.3 (https://www.R-project.org/). The mean value of Papuan-related ancestry proportion at each SNP among Tongan subjects was calculated and plotted across the genome using R package “ggplot2” version 3.1.1 [64]. The list of NCBI RefSeq genes in the detected regions was downloaded from the UCSC Table Browser and implemented in the UCSC Genome Browser [23, 25].

The ELAI analysis was also conducted assuming Aboriginal Taiwanese (Ami and Atayal, n = 35) [30, 46, 48] as a proxy for Asian-related ancestors. The analysis was performed on the dataset consisting of 49,523 autosomal SNPs with the same parameters as above. In addition, to examine the effect of recent European contact, the ELAI analysis was conducted with three-way admixed model assuming CHB, Gidra and CEU (n = 40) as a proxy for Asian-, Papuan- and European- related ancestry, respectively. The analysis was performed on the dataset consisting of 198,803 autosomal SNPs with the same parameters as above.

### Coalescent-based simulation

Coalescent simulations under the assumption of selective neutrality were performed to address whether genetic drift alone could produce similar patterns of admixture across the genome to the ones observed in Tongan subjects. Coalescent-based simulations were performed using the R package “scrm” version 1.7.3.1 [58]. To reproduce the population history of Gidra, CHB and Tonga, a simple population history was assumed based on a single dispersal model into Asia [33, 35, 41] was assumed as described below. First, two subpopulations (Anc1 and Anc2) diverged from one ancestral population 1667 generations ago, which corresponds to 50,000 years ago when generation time is 30 years. Next, subpopulations diverged from Anc1 and Anc2, respectively, and admixed with each other 100 generations ago (3000 years ago). The descendants of Anc1 and Anc2 were regarded as Gidra and CHB, respectively, and the admixed population was regarded as the Tongan (Polynesians) population. Segregating sites within a 1 Mb-long sequence were sampled 3000 times (i.e., 3 Gb long) for 48, 46 and 90 chromosomes from hypothetical Gidra, Tongan and CHB populations, respectively. The mutation rate and recombination rate were set as 1.2 × 10^–8^/ base/generation and 1.3 × 10^–8^/base/generation [3, 29], respectively. The admixture rate in the simulation was given from the mean proportions of Papuan-related ancestry and Asian-related ancestry estimated in the ELAI analysis. The genotype data for 24, 23 and 45 individuals from the hypothetical Gidra, Tongan and CHB populations were generated from the sequences obtained. Considering the SNP ascertainment bias observed in real data, 162,358 SNPs, which exhibited the similar distribution of minor allele frequencies in the real data, were randomly extracted from the simulated genotype data. The above coalescent simulations were performed several times for various population sizes (N = 250, 500, 750, 1,000, 5,000 and 10,000 for each population). Since the mean and SD of Papuan-related ancestry estimated from simulation data for N = 1,000 were most similar to those of real data (Mean = 24.0%, SD = 10.3%), we therefore set a population size of 1,000 for each population in the following analysis. The R code for coalescent simulation under the assumption of selective neutrality is provided in Additional file 2.

To evaluate the family-wise error rate (FWER) and false discovery rate (FDR) of our approach, we performed 100 independent runs of coalescent-based simulations and subsequent ELAI analyses with the same settings as described above. The number of independent genomic regions that exceeded 5 SD from the mean was counted for each simulation run. Here, the mean and SD of Papuan-related ancestry were determined in each run.

### Linkage disequilibrium analysis

To identify a tag SNP for rs17822931-C derived from Papuan-related ancestors, the LD (*D*’ and *r*^2^) of rs17822931 with other SNPs in the franking region of the *ABCC11* was evaluated in 14 Papuans from Simons Genome Diversity Project [33] using Haploview 4.1 [5]. Then, an SNP, rs12445647, which was in strong LD with rs17822931 in SGDP Papuans (*D*’ = 1 and *r*^2^ = 1) and observed in high frequency in Papuans but low frequency in populations from the 1000 Genomes Project Phase 3 [1], was selected as a tag SNP. The LD of rs17822931 with rs12445647 was evaluated in each population of YRI, CEU, CHB, JPT, CHS, CDX, and KHV in the 1000 Genomes Project Phase 3 (1000 Genomes Project Consortium et al. 2015) using LDlink [32].

### Genotyping rs17822931 and rs12445647 in Oceanian populations

Two SNPs, rs17822931 and rs12445647, were genotyped by the TaqMan assay for a total of 616 adult subjects (18 years old or older) from four Oceanian populations: Tongan (n = 174), Munda (n = 170), Gidra (n = 165) and Rawaki (n = 107). Munda people were AN-speaking Melanesians in the New Georgia Islands in the western part of the Solomon Islands. Rawaki village was also located in the Solomon Islands but the inhabitants were regarded as AN-speaking Micronesians as they had migrated there from the overpopulated Gilbert Islands (Kiribati) in the 1960s [40]. Blood sampling was conducted after obtaining informed consent from each subject. Genomic DNA was extracted from peripheral blood using a QIAamp Blood Kit (Qiagen, Hilden, Germany). The LD of rs17822931 with rs12445647 was evaluated in each Oceanian population using Haploview 4.1 [5].

### Approximate Bayesian computation for estimation of selection coefficient

The approximate Bayesian computation was used to estimate the selection coefficient (*s*) for rs17822931-C. We used a forward-time simulation assuming the relative fitness of the CC, CT, and TT genotypes at rs17822931 to be 1, 1-*s*, and 1–2* s*, respectively. The change in allele frequency of rs17822931-C was modeled as follows: the expected allele frequency of rs17822931-C at generation *t* + 1 is given by

$$p_{t + 1} = \frac{{p_{t}^{2} + p_{t} \left( {1 - p_{t} } \right)\left( {1 - s} \right)}}{{p_{t}^{2} + 2p_{t} \left( {1 - p_{t} } \right)\left( {1 - s} \right)\left( {1 - p_{t} } \right)^{2} \left( {1 - 2s} \right)}},$$

where *p*_*t*_ is the allele frequency of rs17822931-C in a Polynesian population at generation *t* since the admixture of Papuan-related and Asian-related ancestors. Assuming that the population size, *N*, is constant, *p*_*t*_ is expressed by *i*_*t*_^*/*^2N, where *i*_*t*_ is the number of copies of rs17822931-C at generation *t*. The number of copies of rs17822931-C at generation *t* + 1 follows the binomial distribution:

$$\Pr ob\left( {i_{ + 1} {|}p_{t} } \right) = \left( {_{{i_{t + 1} }}^{2N} } \right)p_{t + 1}^{i_t + 1} \left( {1 - p_{t + 1} } \right)^{2N - i_{t + 1}}$$

In the computer simulation, *i*_*t+1*_ is generated as a random number based on *P*_*t*_. The initial allele frequency of rs17822931-C in a Polynesian population soon after admixture was given based on the present allele frequencies in Papuan (Gidra) and Asian (CHB) populations (i.e. 0.915 and 0.049, respectively) and the admixture proportion estimated by ELAI analysis (i.e. 0.246 for Papuan-related ancestry and 0.754 for Asian-related ancestry) as follows: 0.915*0.246 + 0.049*0.754 = 0.26. The population size, *N*, was set as 1,000. In each simulation run, the value of *s* was randomly generated using a uniform distribution in the range (0, 1). The value of *s* was recorded only when the allele frequency of rs17822931-C after 100 generations, corresponding to 3000 years, fell within ±5% of the observed allele frequency in the present Tongan population (i.e. 0.722 to 0.798). The mean and 95% credible interval of *s* were calculated for 10,000 successful runs. The computer simulation mentioned above was implemented in R 3.5.3. The R code for approximate Bayesian computation with forward simulation is provided in Additional file 3.

### Comparison of the expression level of *ABCC11* between haplotypes

The expression level of *ABCC11* was compared between haplotypes harboring rs12445647-T and rs17822931-C and harboring rs12445647-T and rs17822931-C using publicly available data: genotype data of the 1000 Genomes Project Phase 3 populations [1] and microarray data of the HapMap3 populations [13, 18, 59] obtained from the ArrayExpress database at EMBL-EBI (www.ebi.ac.uk/arrayexpress) under accession number E-MTAB-264. A total of 217 unrelated subjects with the rs17822931-CC genotype commonly included in the two datasets were used for a regression analysis, where the independent variable of rs12445647 was coded as the number of copies of rs12445647-T (i.e., GG = 0, GT = 1, TT = 2). The 217 subjects belonged to CHB, GIH, JPT, LWK, MEX, MKK and YRI in the 1000 Genomes Project.

## Supplementary Information

**Additional file 1.** Additional figures.

**Additional file 2.** R code for coalescent simulation under the assumption of selective neutrality.

**Additional file 3.** R code for approximate Bayesian computation with forward simulation.

## Data Availability

The data newly created in this study are available on request from the corresponding author. The data are not publicly available due to privacy or ethical restrictions.

## Abbreviations

SD: Standard deviation
xMHC: Extended major histocompatibility complex
ABCC11: ATP-binding cassette transporter sub-family C member 11
AN: Austronesian
HLA: Human leukocyte antigen
PCA: Principal component analysis
CHB: Han Chinese population of Beijing
ELAI: Effective Local Ancestry Inference
FDR: False discovery rate (FDR)
FWER: Family-wise error rate
LD: Linkage disequilibrium
