# Supplementary material for: Admixture with indigenous people helps local adaptation: admixture-enabled selection in Polynesians
Source: BMC Ecol Evol. 2021 Sep 22;21:179. doi: 10.1186/s12862-021-01900-y (PMC8456657; doi:10.1186/s12862-021-01900-y)
Supplement: Supplementary file 1 — Additional file 1. Additional figures. [file 12862_2021_1900_MOESM1_ESM.docx]

## Additional file 1.

**Supplementary figures.**

**Title: Admixture with indigenous people helps local adaptation: admixture-enabled selection in Polynesians**

**Authors: Isshiki et al.**

**
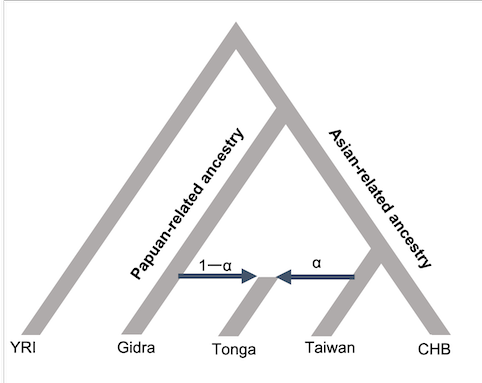
**

**Figure S1. A phylogeny used for f4-ratio estimation.**

CHB and Taiwanese forms a clade and Gidra and YRI are increasingly distant outgroups in this phylogeny. The proportion of Asian-related ancestry in Tonga was α.

**
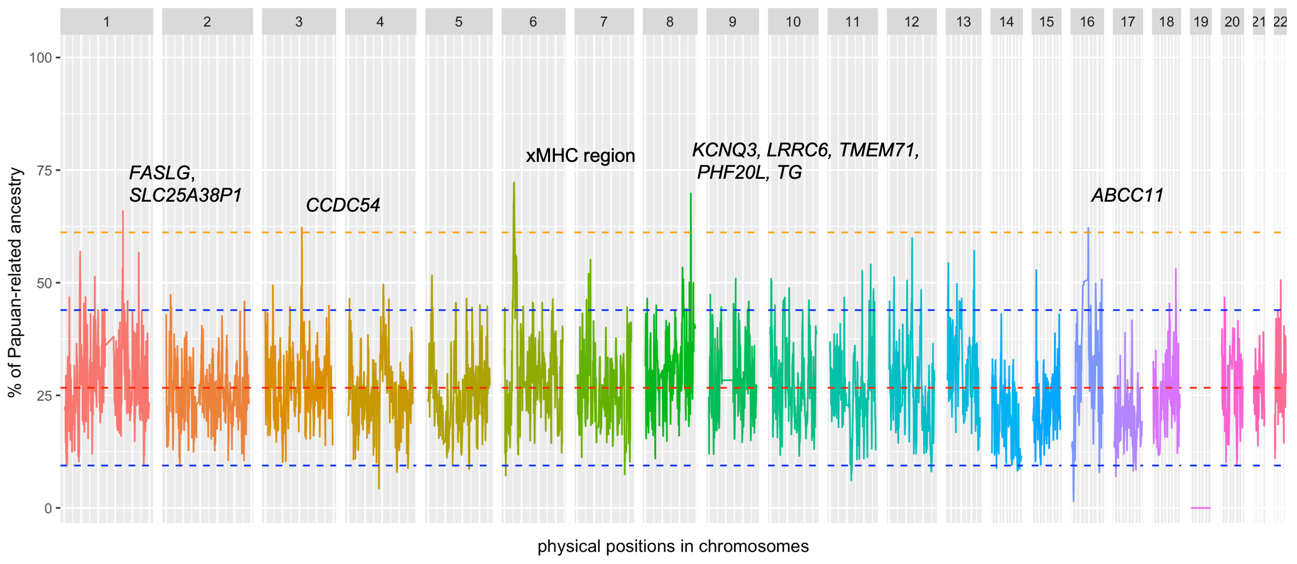
**

**Figure S2. Proportion of Papuan-related ancestry across Tonga genomes assuming Aboriginal Taiwanese as Asian-related ancestors**

Each color represents a different chromosome. Chromosome 19 could not be estimated because of the low SNP density. Red dashed line represents the genome-wide mean. Blue and orange dashed lines represent 2 SD and 4 SD deviations from the mean, respectively. Mean = 26.7 %, SD = 8.63%. The names of the nearest genes or region of the genomic regions outside 4 SD were written in black.

**
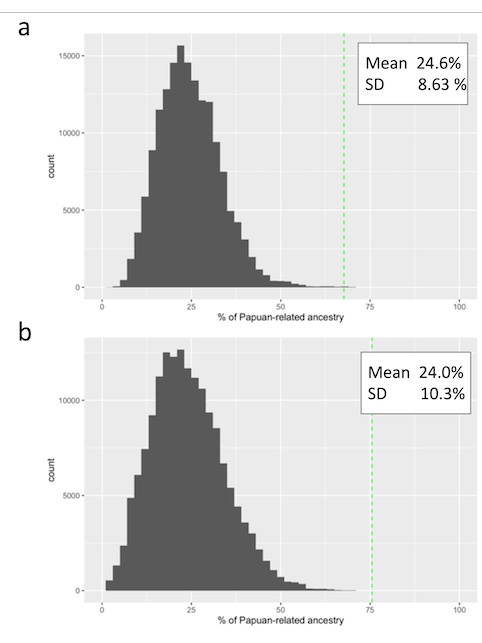
**

**Figure S3. Distribution of Papuan-related ancestry proportions estimated from (a) real data and (b) simulation data**

Green dashed line represents 5 SD deviations from the mean.

**
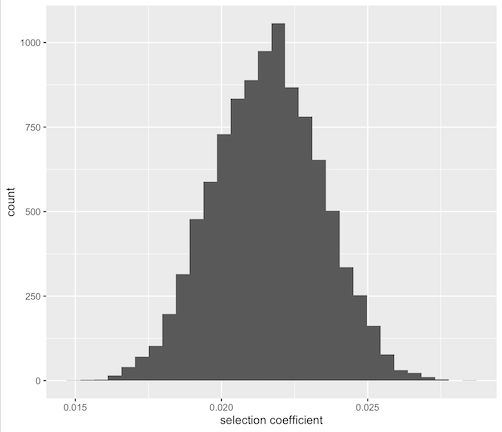
**

**Figure S4. Distribution of selection coefficient for rs17822931-C in Tongans**

Frequency distribution of *s* accepted in simulation runs. The mean and 95% credible interval of *s* are 0.0216 and 0.0180-0.0252, respectively.

**
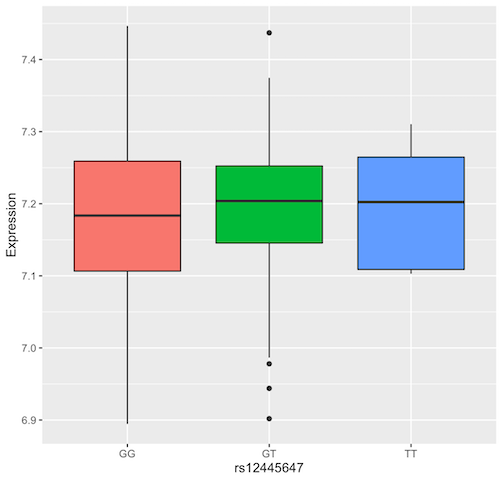
**

**Figure S5. rs12445647 genotypes and the mRNA expression level of the *ABCC11***

The association between rs12445647 genotypes and mRNA expression level was examined for 217 unrelated rs17822931-CC subjects. No significant association was examined.
